# B-1 Cells May Drive Macrophages Susceptibility to *Trypanosoma cruzi* Infection

**DOI:** 10.3389/fmicb.2019.01598

**Published:** 2019-07-09

**Authors:** Raphael Francisco Dutra Barbosa da Rocha, Isabel Ferreira LaRocque-de-Freitas, Angelica Fernandes Arcanjo, Jorgete Logullo, Marise Pinheiro Nunes, Celio Geraldo Freire-de-Lima, Debora Decote-Ricardo

**Affiliations:** ^1^Instituto de Veterinária, Universidade Federal Rural do Rio de Janeiro, Rio de Janeiro, Brazil; ^2^Instituto de Biofísica Carlos Chagas Filho, Universidade Federal do Rio de Janeiro, Rio de Janeiro, Brazil; ^3^Instituto Oswaldo Cruz, FIOCRUZ, Rio de Janeiro, Brazil

**Keywords:** B-1 cells, B-1CDP cells, *Trypanosoma cruzi*, macrophages, susceptibility

## Abstract

B-1 cells can directly and indirectly influence the immune response. These cells are known to be excellent producers of natural antibodies and can secrete a variety of immunomodulatory molecules. They are also able to differentiate into B-1 cell-derived phagocytes (B-1CDP). B-1 cells can modulate macrophages to become less effective, and B-1CDP cells are more susceptible in infection models. In this work, we investigated the microbicidal ability of these cells in *Trypanosoma cruzi* infection *in vitro*. The results show that macrophages from BALB/c mice are more susceptible to infection than macrophages from XID mice. The resistance observed in macrophages from XID mice was abolished in the presence of B-1 cells, and this event seems to be associated with IL-10 production by B-1 cells, which may have contributed to the decrease of NO production. Additionally, B-1CDP cells were more permissive to intracellular *T. cruzi* infection than peritoneal macrophages. These findings strongly suggest that B-1 cells and B-1CDP cells have a potential role in the persistence of the parasite in host cells.

## Introduction

B-1 cells are a cellular subgroup that is present within the B cell compartment. This population differs in several aspects of the majority of the B-cell population, which are known as B-2 or conventional B cells. B-1 cells appear early during embryogenesis in comparison to B-2 cells (Yoshimoto et al., 2011). In addition, they are found in different locations. While B-2 is found in peripheral blood and lymphoid organs, B-1 cells populate serous cavities such as the peritoneum and pleura and are found in very low percentages in the spleen.

B-1 cells may differ from B-2 cells in the expression of surface markers (Baumgarth, 2017). Initially, murine B-1 cells were identified by the presence of the CD5 molecule (Hayakawa et al., 1983). However, the phenotypic characterization of B-1 cells is currently based on the expression of a set of surface molecules that have made it possible to identify subtypes B-1a and B-1b (Stall et al., 1992). The characterization is based on the combination of the following: markers CD5^+^, CD11b^+^, (Mac-1), CD45RA^lo^, IgM^hi^, IgD^lo^, CD23^–^, CD-19^+^ and CD43^+^ for B-1a; CD5^–^, CD45RA^lo^, IgM^hi^, IgD^lo^, CD23^–^, CD43^+^ and CD-19^+^ for B-1b; and CD5^–^, CD11b^–^, CD45RA^hi^, IgM^lo^, IgD^hi^, CD23^+^ and CD43^–^ for B-2 (Yenson and Baumgarth, 2014).

B-1 cells are known to be excellent producers of natural antibodies, particularly IgM and IgG3, which can bind to both self and non-self-antigens (Baumgarth, 2011). B-2 cells are dependent on foreign antigens and cooperation with T cells to activate and differentiate into antibody-producing cells. However, B-1 cells evolve in these steps spontaneously and become natural antibody-producing cells (Baumgarth, 2017). In addition to the production of antibodies, B-1 cells are able to secrete a variety of immunomodulatory molecules, such as IL-10. After stimulation with LPS, they produce IL-3 and GM-CSF, among other molecules, which are able to modulate both the acute and chronic inflammatory response (Chousterman and Swirski, 2015). In addition, they play a role in efficient antigen presentation to T cells with the expression of CD80 and CD86 co-stimulatory molecules in the B-1a subpopulation (Hirose et al., 2017). Thus, they are also able to influence the differentiation of T lymphocytes into the Th17 profile, which are closely involved in inflammatory responses (Zhong et al., 2007).

B cell deficiency has often been associated with the impairment of immunity against infection. Individuals with X-linked agammaglobulinemia (XLA) have a mutation in the gene that is responsible for Bruton tyrosine kinase (BTK), which involves a failure in antibody production and a high susceptibility to bacterial infections (Simao-Gurge et al., 2017). There is a murine model that exhibits the same impairment and leads to a similar state of susceptibility to that in humans. BALB/c mice that present a mutation in the gene responsible for BTK are called XID mice. When infected, these animals demonstrate a progressive inflammatory response that is mediated by the production of pro-inflammatory molecules and decreased production of IL-10. These characteristics have been observed during infection by *Paracoccidioides brasiliensis* (Popi et al., 2008), Filaria (Mukhopadhyay et al., 1999), Leishmania (Arcanjo et al., 2017), and *Trypanosoma cruzi* (Minoprio et al., 1993).

Our group has recently demonstrated that B-1 cells are also associated with susceptibility in a murine model of leishmaniasis. In visceral leishmaniasis, the splenic production of IL-10 compromises the microbicidal activity of macrophages, but XID mice are more resistant (Arcanjo et al., 2017). In addition, we demonstrated that the PGE2/IL-10 axis is involved in the susceptibility of B-l lymphocyte-derived phagocytes (B-1CDP) in *L. major* infection (Arcanjo et al., 2015).

In *P. brasiliensis* infection, the activation of macrophages and the production of nitric oxide (NO) and hydrogen peroxide seem to be fundamental in the control of fungus growth and dissemination (Popi et al., 2008). It was demonstrated that there is less phagocytosis of yeasts of *P. brasiliensis* by macrophages derived from XID mice co-cultured with B-1 cells in Transwell^®^ system than by cultures of macrophages in the absence of the B-1 cells. This suggests the participation of soluble factors produced by B-1 lymphocytes. The cytokine IL-10 may be thus be important soluble mediator (Popi et al., 2004; Arcanjo et al., 2015, 2017; Gonzaga et al., 2015).

B-1 cells represent the only lymphocytic cell population that differentiates into macrophage-like phagocytes called B-1CDP. Similarly to macrophages, B-1CDP are able to migrate to inflammatory sites (Almeida et al., 2001). Recently, our group demonstrated that B-1CDP cells are more susceptible to *Leishmania major* infection (Arcanjo et al., 2015) and play an important role in the development of murine macrophage resistance in visceral leishmaniasis (Arcanjo et al., 2017).

*T. cruzi* is an intracellular parasite that causes Chagas disease. This pathogen can infect any nucleated cell of a vertebrate host. After cell invasion, the trypomastigote forms differentiate into the amastigote intracellular forms, and replication phase begins. They then transform into trypomastigotes, break the host cells, and become free to infect others cells. The trypomastigote forms can reach the bloodstream and lymphatic system, and the distribution of the organisms gives rise to new outbreaks of infection. Chagas disease also has different phases. The acute phase is characterized by the presence of trypomastigote forms in the blood, and the latency phase or indeterminate phase can last for long periods or for life. Some infected individuals can develop into a chronic phase (Rassi et al., 2010).

*Trypanosoma cruzi* has great ability to infect macrophages by subverting their defense mechanisms (Campo et al., 2016). Although macrophages have several activation mechanisms, they are usually good host cells for *T. cruzi* (Freire-De-Lima et al., 2000; De Souza et al., 2010; Decote-Ricardo et al., 2017; Mendonca et al., 2017). In addition to subverting the microbicidal mechanisms by the parasite, it is possible that endogenous factors such as the production of modulating cytokines are partly responsible for the success of the infection (Dutra et al., 2014; Luna-Gomes et al., 2014; Decote-Ricardo et al., 2017; Mendonca et al., 2017). In *T. cruzi* infection, XID animals exhibit a decrease in IL-10 production and favor the production of IFN-γ and IL-2, which may be determinants in the control of parasitism (Minoprio et al., 1993). This information suggests an increased susceptibility of macrophages in the presence of B-1 cells. Based on this, we investigated the modulatory effect of B-1 cells and the susceptibility of B-1CDP on infection by *T. cruzi in vitro*.

## Materials and Methods

### Ethics Statement

This study was carried out in strict accordance with the recommendations in the Guide for the Care and Use of Laboratory Animals of the National Institutes of Health (United States). The protocol was approved by the Committee on the Ethics of Animal Experiments of the Health Science Center of the Federal University of Rio de Janeiro (CEUA-CCS, Permit Number: IBCCF 062/14), and all efforts were made to minimize suffering.

### Animals

BALB/c and BALB XID (B-1 cells deficient) mice of both sexes, aged 6-8 wk, were obtained from the Oswaldo Cruz Institute Animal Care Facility (Fiocruz, Rio de Janeiro, Brazil). The mice were used to obtain B-1 cells and macrophages by peritoneal lavage.

### B-1 and B-1CDP Cells

B-1 cells were obtained from BALB/c mice using the protocol previously described (Abrahao et al., 2003). Macrophages were harvested by peritoneal lavage of BALB/c or BALB/c XID mice using cold DMEM medium (Gibco, Life Technologies). The total population of cells from the peritoneum was plated into 25 cm^2^ tissue culture flasks (Corning) and incubated at 37°C in a 5% CO_2_ atmosphere for 2 h. The non-adherent cells were discarded, and DMEM medium containing 2 mmol/L glutamine, 50 μmol/L 2-ME, 10 μg/mL of gentamicin, 1 mmol/L sodium pyruvate, and 100 μmol/L MEM non-essential amino acids plus 10% fetal calf serum (FCS) was added to the adherent monolayer. The cultures were maintained for 5 days without changing the medium under the conditions described above. As indicated by flow cytometry, the enriched 5 day cultured B-1 cells showed 87.8% purity and a viability of 98.3% (Supplementary Figure S1), whereas the adherent cells represented an enriched macrophage population. The portion of the non-adherent cells (B-1), were also used for differentiation into B-1CDP. After this, the cells were cultured for another 3 days on coverslips in the bottom of 6 well plates for differentiation in B-1CDP in the absence of any stimulus as previously described (Almeida et al., 2001). After this time in culture B-1CDP, adherent to the glass surface were removed from the substrate by ice-cold phosphate-buffered saline. Cells were counted, added (2 × 10^5^) to glass cover slips inserted in 24-well tissue culture plates. Peritoneal macrophages cultures were made as above described using adherent cells from the peritoneal cavity of BALB/c. Peritoneal macrophages were counted, added (2 × 10^5^) to glass coverslips inserted in 24-well tissue culture plates.

### Phenotypic and Viability Analysis of Non-adherent Fractions of Cell Cultures by FACS

Cells from non-adherent fractions of the 5 days culture described above were collected, and were stained with the following antibodies: floating cells were stained with allophycocyanin (APC) labeled anti-mouse CD19 (clone 1D3) and fluorescein-isothiocyanate (FITC) labeled anti-mouse IgM (clone G155-228) (Pharmingen, San Diego, CA, United States). The cells were incubated with blocking buffer (FcBlock CD16/CD32 – eBioscience) for 30 minutes at room temperature and all washing steps were performed with phosphate-buffered saline containing 3% bovine serum albumin and 0.02% of sodium azide. After staining for cell surface markers, isotonic propidium iodide (PI) (Sigma, ST Louis, MO, United States) solution (10x) was added to each tube (final concentration 1x) and incubated for 1 minute. Data were acquired (10,000 events), evaluated on FACSCalibur^TM^ cytometer, and analyzed using Cell-Quest^®^ software (BD Biosciences, Heidelberg, Germany). Our lymphocyte gate was made in an enriched B-1 cells culture. Dot plots are representative of the analysis of cell size and complexity and the CD19 and IgM expression.

### Parasites and Infection

Chemically induced metacyclic trypomastigotes forms of *T. cruzi* clone Dm28c, obtained as previously described (Contreras et al., 1985) were used to infect murine macrophages. The macrophage was carried out in 24-well plates at a concentration of 2 × 10^5^ macrophages/well and infected overnight with *T. cruzi* at a 5:1 parasite-to-cell ratios in 1 mL of DMEM 10% FCS and incubated at 37°C in 5% CO_2_. In the following day (day 1), monolayers were extensively washed to remove extracellular parasites and cultured with complete culture medium containing 1% Nutridoma (Sigma-Aldrich) instead of FCS. Some cultures were co-cultivated with B-1 cells at a 10:1 B-1-to-macrophages ratios. We stimulated the cultures with 400 ng/mL of lipopolysaccharides from *Salmonella enterica* serotype *typhimurium* (LPS, Sigma-Aldrich) and 1.5 ng/mL of murine IFN-γ (Serotech) 24 h after infection. Some cultures were performed containing glass coverslips for parasite burden evaluation. 3 days after infection, coverslips were washed with HBSS, fixed with methanol, and stained with Diff-Quick (Thermo Fisher Scientific, Waltham, MA, United States). Amastigotes were counted at 100× oil immersion on a (Olympus) microscope. The number of amastigotes was estimated in 100 infected cells per coverslip, and the frequency of infection was compared among six coverslips per time point. The number of viable trypomastigotes released in the supernatants was evaluated 7 and 9 days after infection, using a Neubauer chamber previously described (Nunes et al., 1998).

### Cytokine Determination

Peritoneal macrophages obtained from BALB/c mice and XID (2 x 10^5^ macrophages/well) were seeded in 24-well plates. Some cultures were infected with trypomastigote form of *T. cruzi* overnight. After washing some cultures were stimulated with LPS (400 ng/mL) and IFN-γ (1.5 ng/mL) and co-cultivated or not with B-1 cells. Cell supernatants were collected at 24 h after stimulation for cytokine determination. IL-10, concentration was estimated by the method of sandwich immunoassay (ELISA) according to methodology recommended by the manufacturer (R&D). The optical density was evaluated by reading in a microplate spectrophotometer (Versamax Microplates Reader Molecular Devices, United States), with filter of 405 nm.

### Evaluation of Nitric Oxide

Peritoneal macrophages obtained from BALB/c mice and XID (2 × 10^5^ macrophages/well) were seeded in 24-well plates. Some cultures were infected with trypomastigote form of *T. cruzi* overnight. After washing some cultures were stimulated with LPS (400 ng/mL) and IFN-γ (1.5 ng/mL) and co-cultivated or not with B-1 cells. The nitric oxide (NO) produced was quantified by the presence of nitrite accumulated in the supernatant of cultures using the Griess colorimetric method previously described (Kwon et al., 1989).

### Statistical Analysis

Statistical analysis was performed in the program GraphPad InStat version 3.01 (San Diego, United States). Data were analyzed by the method of Student’s *t*-test. Differences at *p*-Value 0.05 or lower were reported as significant for a given comparison.

## Results

### Interaction With B-1 Cells Regulates Replication of *T. cruzi* in Macrophages From BALB/c and XID Mice

**FIGURE 1:**
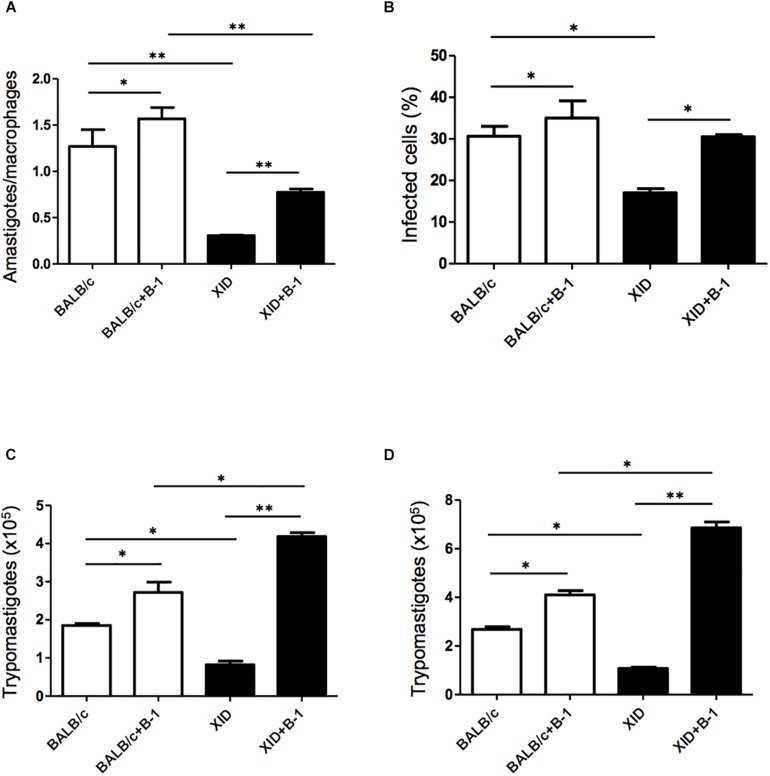
Effect of B-1 cells in co-culture with macrophages infected by *Trypanosoma cruzi*. Peritoneal macrophages from BALB/c (white bars) or from XID mice (black bars) were plated (2 × 10^5^/mL) and infected with trypomastigotes forms of *T. cruzi* Dm28c clone at a 5:1 parasite-to-cell ratios in 1 mL of DMEM 10% FCS and incubated at 37°C in 5% CO_2_. After overnight incubation, the cell culture was washed and some cultures were co-cultivated with B-1 cells at a 10:1 B-1-to-macrophages ratios. After 3 days, cells were stained and amastigotes inside the macrophages were counted under light microscope **(A)** and set the percentage of infected cells **(B)**. To quantify trypomastigotes forms in the supernatants, the cells were infected with trypomastigotes forms of *T. cruzi* Dm28c clone. After overnight incubation, the cell culture was washed and some cultures were co-cultivated with B-1 cells at a 10:1 B-1-to-macrophages ratios for 7 **(C)** and 9 **(D)** days. All cultures were performed in triplicate and bars show the mean + SD. Statistical analysis was performed by *t*-test from representative results of three similar experiment (^*^p < 0.05 and ^∗∗^*p* < 0.01).

Macrophages are crucial to the establishment of infection as a host cell and for the eradication of parasitism in the acute phase (Montalvao et al., 2018; Volpini et al., 2018). In order to evaluate the susceptibility of the macrophages from BALB/c or XID mice, we plated the macrophages and infected them with *T. cruzi* Dm 28c. After 3 days, we observed a greater number of intracellular amastigotes in macrophages from BALB/c than those from XID mice (Figure 1A), and the percentage of infected cells was also higher in BALB/c macrophages (Figure 1B).

After seven (Figure 1C) and nine (Figure 1D) days of infection, we also observed a great release of trypomastigote forms in these macrophages. Macrophages from BALB/c mice were extremely more susceptible to *T. cruzi* infection than XID macrophages. However, macrophages from BALB/c cultured with B-1 cells showed an increase in the number of replicative forms of amastigotes and the release of trypomastigotes (Figures 1A,C,D). Furthermore, macrophages from XID mice cultured with B-1 cells from BALB/c became more susceptible to infection. In this condition, the presence of B-1 cells increased the number of intracellular amastigotes, the released trypomastigotes in the cellular supernatant, and the percentage of infected cells. The results suggest that B-1 cells favor intracellular parasitism and possibly negatively modulate the trypanocidal activity of infected macrophages.

### B-1 Cells Contribute to Marked Reduction in Nitric Oxide Production

The production of NO is a crucial strategy in the control against many infectious diseases (Watanabe Costa et al., 2016; Sanmarco et al., 2017). Macrophages stimulated with classic activators such as LPS or CPG DNA are able to induce the production of large amounts of NO (Gordon, 2003). To determine whether B-1 cells could modulate the production of NO, macrophages from BALB/c and XID mice were infected with *T. cruzi* and co-cultured in the presence and absence of B-1 cells. After 24 h, the NO production was measured.

**FIGURE 2:**
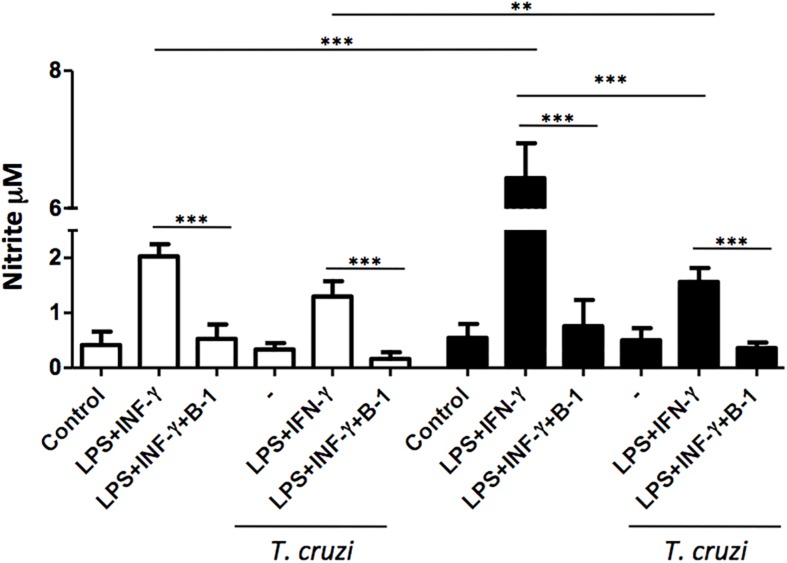
The production of nitric oxide in macrophage: B-1 cell co-cultures. Peritoneal macrophages from BALB/c (white bars) or from XID mice (black bars) were plated (2 × 10^5^/mL) and infected with trypomastigotes forms of *T. cruzi* Dm28c clone at a 5:1 parasite-to-cell ratios in 1 mL of DMEM 10% FCS and incubated at 37°C in 5% CO_2_. After overnight incubation, the cell culture was washed and some cultures were co-cultivated with B-1 cells at a 10:1 B-1-to-macrophages ratios and stimulate or not with LPS (400 ng/mL) and IFN-γ (1.5 ng/mL). After 24 h, the NO was measured in the supernatants of the cultures by the Griess method. All cultures were performed in triplicate and bars show the mean + SD. Statistical analysis was performed by *t*-test from representative results of three similar experiment (^∗∗^*p* < 0.01 and ^∗∗∗^*p* < 0.001).

In the absence of B-1 cells, stimulated macrophages from XID mice produced large amounts of NO in comparison with macrophages from BALB/c mice. We also observed that stimulated and infected XID macrophages show a reduction in NO production. Additionally, when macrophages were co-cultured with B-1 cells, there was a significant reduction in NO production in both XID and BALB/c macrophages (Figure 2). These results strongly suggest a possible modulatory role of B-1 cells on the NO production by macrophages.

### IL-10 Production Is Dependent on B-1 Cells

B-1 cells are known to induce an anti-inflammatory and anti-immunogenic response, which is mediated in part by their induction of active IL-10 (Barbeiro et al., 2011; Chousterman and Swirski, 2015). IL-10 is a cytokine that is capable of modulating the microbicidal activity of macrophages and inducing a profile that is more permissive to intracellular infections (Arcanjo et al., 2017; Aziz et al., 2017; Smith and Baumgarth, 2019). Thus, after confirming that there was a reduction in NO production in the presence of B-1 cells, we evaluated whether IL-10 production by B-1 cells could be involved in the modulation of NO production. We conducted experiments where cultures of macrophages from BALB/c or XID mice were infected with *T. cruzi* and cultured in the presence or absence of B-1 cells.

**FIGURE 3:**
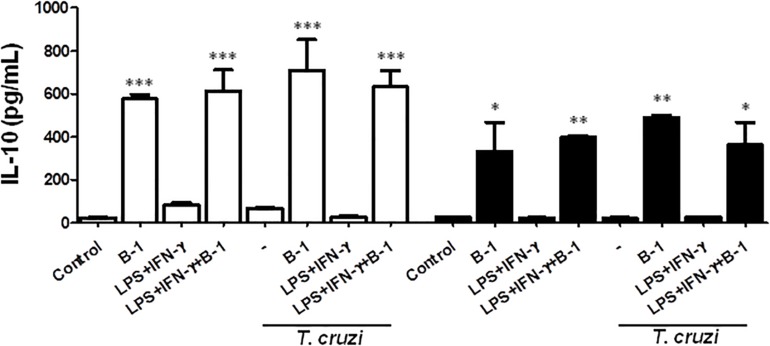
The production of the IL-10 in macrophage: B-1 cell co-cultures. Peritoneal macrophages from BALB/c (white bars) or from XID mice (black bars) were plated (2x10^5^/mL) and infected with trypomastigotes forms of *T. cruzi* Dm28c clone at a 5:1 parasite-to-cell ratios in 1 mL of DMEM 10% FCS and incubated at 37°C in 5% CO_2_. After overnight incubation, the cell culture was washed and some cultures were co-cultivated with B-1 cells at a 10:1 B-1-to-macrophages ratios and stimulate the cultures with LPS (400 ng/mL) and IFN-γ (1.5 ng/mL). After 24 h, the supernatant was collected and IL-10 was measured by ELISA. The test detection limits for IL-10 were 15.6–1000 pg/mL. All cultures were performed in triplicate and bars show the mean + SD. Statistical analysis was performed by *t*-test from representative results of three similar experiment (^*^p < 0.05, ^∗∗^*p* < 0.01, and ^∗∗∗^*p* < 0.001).

After 24 h, the supernatant was collected, and the IL-10 cytokines were measured. The results indicated elevated levels of IL-10 produced by B-1 cells alone, and in the culture of macrophages in the presence of the B-1 cells. Both BALB/c and XID macrophages cultured in the presence of B-1 cells showed increased levels of IL-10 independent of whether they were infected or not (Figure 3). These assays suggest that B-1cells are a very important source of IL-10, which is able to modulate macrophages to become more susceptible to infection by *T. cruzi*.

### B-1CDP Cells Are More Susceptible to Infection With *T. cruzi* Than Peritoneal Macrophages

**FIGURE 4:**
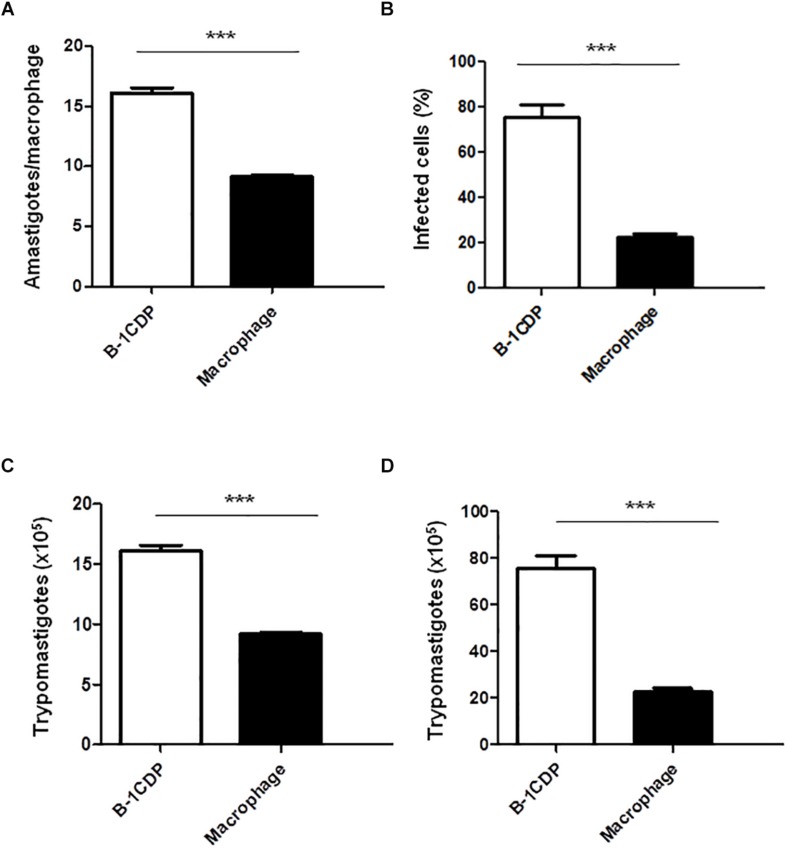
Susceptibility of B-1CDP to *Trypanosoma cruzi*. B-1CDP (black bars) or peritoneal macrophages from BALB/c (white bars) were plated (2 × 10^5^/mL) and infected with trypomastigotes forms of *T. cruzi* Dm28c clone at a 5:1 parasite-to-cell ratios in 1 mL of DMEM 10% FCS and incubated at 37°C in 5% CO_2_. After overnight incubation, the cell culture was washed and incubated with DMEM 10% FCS and at 37°C in 5% CO_2_. After 3 days, cells were stained and amastigotes inside the macrophages were counted under light microscope **(A)** and set the percentage of infected cells **(B)**. To quantify trypomastigotes forms in the supernatants, the cells were infected with trypomastigotes forms of *T. cruzi* Dm28c clone. After overnight incubation, the cell culture was washed and incubated with DMEM 10% FCS and at 37°C in 5% CO_2_ for 7 days **(C)** and 9 days **(D)**. All cultures were performed in triplicate and bars show the mean + SD. Statistical analysis was performed by *t*-test from representative results of three similar experiment (^∗∗∗^*p* < 0.001).

B-1 cells have the unique ability to differentiate into macrophage-like cells (Almeida et al., 2001) called B-1CDP. In models of *L. major* infection, they are more susceptible than peritoneal macrophages (Arcanjo et al., 2015, 2017). In order to verify the susceptibility of B-1CDP cells to *T. cruzi* infection, we cultured macrophages obtained from BALB/c mice and B-1CDP differentiated from B-1 cells and performed the infection with *T. cruzi* Dm 28c. After 3 days, we observed a greater number of intracellular amastigotes in B-1CDP than peritoneal macrophages (Figure 4A). We also observed that the percentage of infected cells was higher in BALB/c macrophages (Figure 4B). To quantify the release of trypomastigote forms, the cultures were maintained for 9 days and revealed a much greater release of trypomastigote forms by B-1CDP cells than by peritoneal macrophages (Figures 4C,D). Our results suggest that B-1CDP cells present a greater susceptibility to *T. cruzi* infection than peritoneal macrophages.

## Discussion

B-1cells were initially known to have an important function as a source of natural antibodies (Baumgarth, 2011). However, we now know through several reports that its functions go far beyond the production of antibodies. B-1 cells are producers of a variety of molecules, such as IL-10, IL-3, and GM-CSF (Chousterman and Swirski, 2015), which are capable of exerting influence on other cell populations and thus may influence the susceptibility or resistance in models of infection (Minoprio et al., 1993; Popi et al., 2008; Arcanjo et al., 2017). These immunomodulatory effects were observed in different models involving infection and inflammation (Oliveira et al., 2010; Arcanjo et al., 2015; Gambero et al., 2016; Aziz et al., 2017). Our group has recently demonstrated that IL-10 production contributes to susceptibility in models of *L. major* infection (Arcanjo et al., 2015, 2017) and *Leishmania amazonensis* (Firmino-Cruz et al., 2018).

In the present work, we showed that macrophages from BALB/c mice are more susceptible to *T. cruzi* infection than macrophages obtained from XID mice, which was evidenced by the high number of infected cells, the number of amastigote forms within the macrophages, and the release of trypomastigote forms. However, macrophages from XID mice became more susceptible when co-cultured with B-1 cells from BALB/c mice. The presence of B-1 cells was a determinant for the increased susceptibility of BALB/c macrophages and for the breakdown of the resistance of macrophages from XID mice. These results resemble those observed in a study on *L. major* infection, where we previously demonstrated that macrophage susceptibility was increased in the presence of B-1 cells (Arcanjo et al., 2017).

The activation of macrophages may be a determining factor for the elimination of an intracellular pathogen. Activation increases microbicidal activity, and NO production can be crucial in this process. On the other hand, the suppression of this molecule may contribute to the susceptibility and success of the parasite (Watanabe Costa et al., 2016; Sanmarco et al., 2017). For this reason, we evaluated if the production of NO was being affected by the presence of B-1 cells. Macrophages from XID mice exhibit extremely high NO production in comparison to macrophages from BALB/c mice. However, in the presence of B-1 cells, there was a marked reduction of NO, which suggests that B-1 cells may influence the ability of macrophages to produce NO.

Many studies have already reported that B-1 cells a role in the production of IL-10 and are able to modulate other types of cells (De-Gennaro et al., 2009; Geraldo et al., 2016; Liu et al., 2016). IL-10 is a cytokine that can act on different types of cells and leads to the inhibition of several molecules that have an important role in the activation of macrophages and NO (Ouyang et al., 2011; Liu et al., 2016). NO has trypanocidal activity and contributes to the arginase pathway by participating in the activity of ornithine decarboxylase (Freire-De-Lima et al., 2000; Sanmarco et al., 2017). An imbalance between NO and arginase production may be a determinant for the resistance against and susceptibility to *T. cruzi* infection in a murine model (Felizardo et al., 2018).

The reduction in NO production by macrophages co-cultivated with B-1 cells could be a result of the action of IL-10 on the macrophages. Taking this into account, we evaluated the production of IL-10 under the same conditions. The results revealed that the production of NO was significantly reduced in the presence of high concentrations of IL-10. This corroborates our previous work, which showed increased intracellular parasitism of macrophages by *L. major* in the presence of B-1 cells (Arcanjo et al., 2017). Our findings are in accordance with previous studies demonstrating that mice with B cell deficiency may be resistant to infection by *T. cruzi* (Minoprio et al., 1991, 1993).

B-1 is the only type of lymphocyte with the ability to differentiate into phagocytes. After differentiation into B-1CDP, they acquire morphological and functional characteristics similar to those of phagocytes (Almeida et al., 2001). Furthermore, they may migrate to sites of inflammation and help in healing (Popi et al., 2009; Oliveira et al., 2010) or in situations of infection that contribute to the success of the pathogen (Popi et al., 2009; Arcanjo et al., 2015). It has also been demonstrated that B-1CDP cells are more susceptible to *L. major* infection and that the PGE2/IL-10 axis contributes to susceptibility (Arcanjo et al., 2015).

To demonstrate whether B-1CDP phagocytes could be more permissive to *T cruzi* infection, we induced the differentiation of B-1 cells into B-1CDP and compared them to peritoneal macrophages that were also infected. We observed that B-1CDP cells are much more permissive to infection than peritoneal macrophages in all aspects analyzed. The B-1CDP cells presented a higher percentage of infected cells with a greater number of intracellular amastigote forms, which resulted in the release of larger amounts of trypomastigote forms in culture. Similar results were previously reported by our group and demonstrated that B-1CDP phagocytes produce high concentrations of IL-10 when infected or not infected by *L. major.* To consolidate the role of IL-10 in susceptibility to infection, we observed that B-1CDP from IL-10 KO mice showed decreased susceptibility to *L. major*.

The present results point to the important immunomodulatory role of IL-10 in macrophage activity against *T. cruzi* infection. The data strongly suggest that IL-10 plays an inhibitory role in NO production and thus favors parasitism. In addition, B-1CDP phagocytes were highly susceptible to *T. cruzi* infection. Based on the fact that they retain the ability to produce IL-10, they appear to remain under the immunomodulatory effects of this cytokine and thus show susceptibility as well. Popi et al. (2009) demonstrated a similar result using a model of *Coxiella burnetti in vitro*. They found that macrophages from XID mice that are deficient in B-1 cells presented more resistance to intracellular infection by *C. burnetti* than peritoneal macrophages from BALB/c mice. Based on our results and the information reported in the literature, we believe that IL-10 produced by B-1CDP cells is the key to understanding their increased susceptibility to infection.

In summary, we suggest that B-1 cells can contribute to the susceptibility as producers of cytokine, particularly IL-10 or after differentiating into B-1CDP and becoming excellent host cells. In both ways, the cells can favor the detrimental effects of the parasite on the host. The aim of our experiments was only *in vitro* analysis, which means that *in vivo* experiments could better clarify these phenomena and point out other relevant factors.

## Data Availability

All datasets generated for this study are included in the manuscript and/or the Supplementary Files.

## Ethics Statement

This study was carried out in strict accordance with the recommendations in the Guide for the Care and Use of Laboratory Animals of the National Institutes of Health (United States). The protocol was approved by the Committee on the Ethics of Animal Experiments of the Health Science Center of the Federal University of Rio de Janeiro (CEUA-CCS, Permit Number: IBCCF 062/14), and all efforts were made to minimize suffering.

## Author Contributions

DD-R, CF, and RR conceived and designed the experiments. RR, IL-d-F, AA, and JL performed the experiments. DD-R, CF, and MN analyzed the data. DD-R and CF wrote the manuscript.

## Conflict of Interest Statement

The authors declare that the research was conducted in the absence of any commercial or financial relationships that could be construed as a potential conflict of interest.

## Supplementary Material

FIGURE S1 Viability and percentage of B-1 cells after culture. **(A)** Schematic representation of gate strategy after 5 days of culture. Expression of CD-19 and propidium iodide (PI) were evaluated on lymphocyte to determine the percentage of viable cells that was 98.3% (upper left quadrant). In order to establish the percentage of B-1 cells, we verified the expression of CD-19 and IgM in 87.8% of the cells (right upper quadrant). **(B)** Graph represents percentage of viable B-1 (CD-19^+^ PI^–^). Statistical analysis was performed by *t*-test from representative results of three similar cultures (^∗∗∗^*p* < 0.001).
