# Supplementary figures and images for: B-1 Cells May Drive Macrophages Susceptibility to Trypanosoma cruzi Infection
Source: Front Microbiol. 2019 Jul 9;10:1598. doi: 10.3389/fmicb.2019.01598 (PMC6629875; doi:10.3389/fmicb.2019.01598)

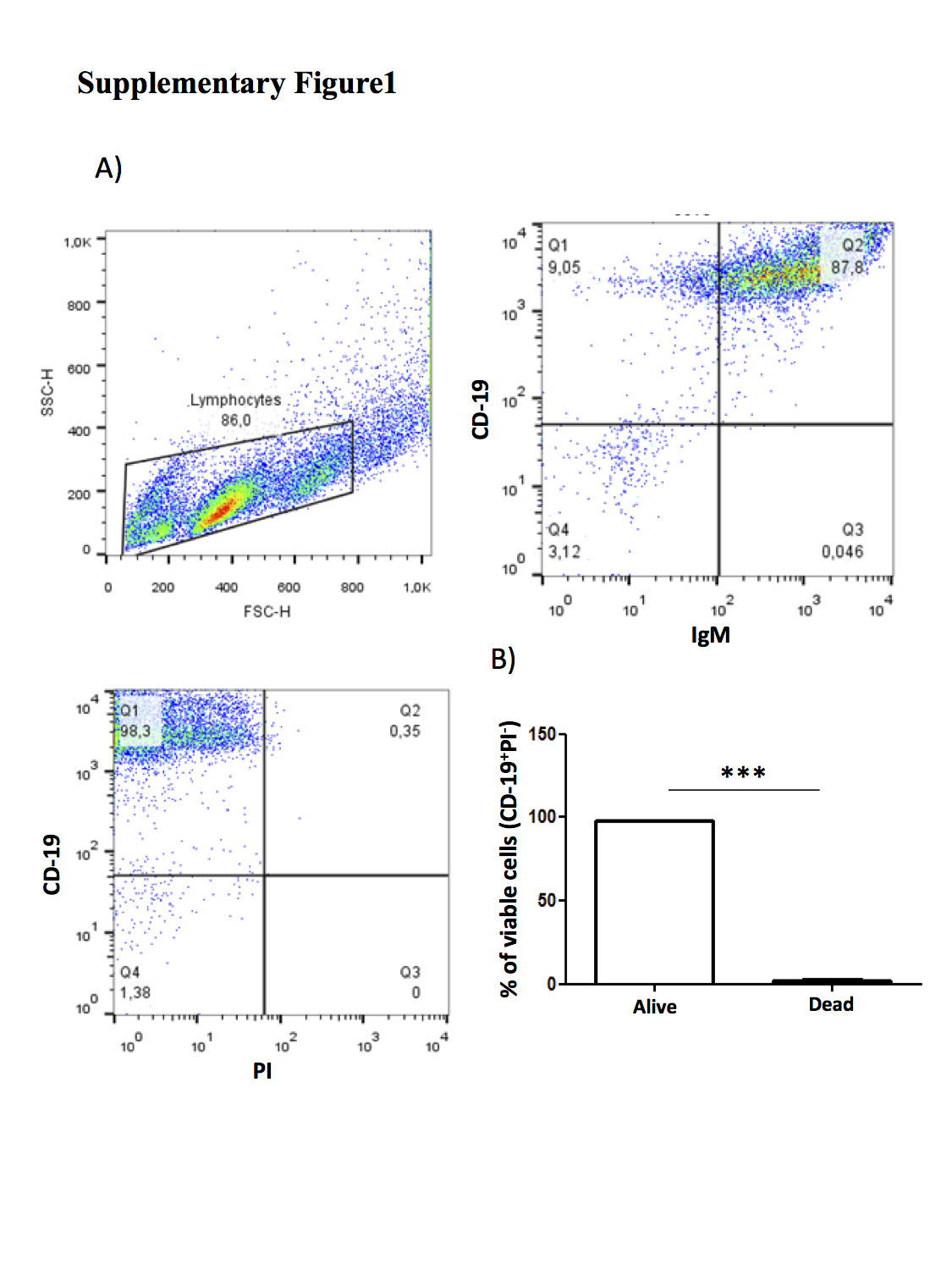

Supplement: FIGURE S1 — Viability and percentage of B-1 cells after culture. (A) Schematic representation of gate strategy after 5 days of culture. Expression of CD-19 and propidium iodide (PI) were evaluated on lymphocyte to determine the percentage of viable cells that was 98.3% (upper left quadrant). In order to establish the percentage of B-1 cells, we verified the expression of CD-19 and IgM in 87.8% of the cells (right upper quadrant). (B) Graph represents percentage of viable B-1 (CD-19+ PI–). Statistical analysis was performed by t-test from representative results of three similar cultures (∗∗∗p < 0.001). [file Image_1.TIFF]
